# Introduction of electronic data capture method using participant-completed online web-based follow up questionnaire in mail-based study achieves expected benefits and positive participant feedback

**DOI:** 10.1186/1745-6215-16-S2-P44

**Published:** 2015-11-16

**Authors:** Jill Barton, Allen Young, Michael Lay

**Affiliations:** 1 University of Oxford, Oxford, UK

## Background

To reduce data processing and postal costs, and to potentially enhance data quality in a large long-term mail-based randomised trial, the ASCEND study team developed a bespoke online web-based version of the standard 6-monthly paper follow-up questionnaire. The form incorporates verification checks and study staff were trained to provide IT support for participants. The key IT challenges included ensuring screen format compatibility with different computer devices and software compatibility with multiple browsers and versions, as well as ensuring data security which was enhanced by using a two factor user authentication scheme. The aim was to assess the impact of providing the option to study participants in a small pilot study.

## Methods

In November 2014, 200 randomly selected participants [aged 46 -89 years] were mailed their paper questionnaire with a flyer giving details of the new web option. Of the 200 participants, 188 [94%] responded with 29 [15% of total responders] completing their questionnaire on-line [aged 46-84 years]. For web forms the data quality was significantly better with reduced data processing time compared with the equivalent paper form. Users provided positive feedback with 8 IT-related queries. A subsequent mailing to the same group of 200 resulted in 39 web responders, suggesting that the uptake may increase by continuing to advertise the option at the time of each mailing coupled with an awareness campaign via the study newsletter.

## Conclusion

These early results provide encouraging evidence for adopting a web-based approach for similar studies where email addresses can be captured at screening.

